# Relaxation Time Estimation from Complex Magnetic Resonance Images

**DOI:** 10.3390/s100403611

**Published:** 2010-04-09

**Authors:** Fabio Baselice, Giampaolo Ferraioli, Vito Pascazio

**Affiliations:** Dipartimento per le Tecnologie, Università degli Studi di Napoli Parthenope, Naples, Italy; E-Mails: fabio.baselice@uniparthenope.it (F.B.); vito.pascazio@uniparthenope.it (V.P.)

**Keywords:** Magnetic Resonance Imaging, relaxation parameter estimation, statistical signal processing

## Abstract

Magnetic Resonance (MR) imaging techniques are used to measure biophysical properties of tissues. As clinical diagnoses are mainly based on the evaluation of contrast in MR images, relaxation times assume a fundamental role providing a major source of contrast. Moreover, they can give useful information in cancer diagnostic. In this paper we present a statistical technique to estimate relaxation times exploiting complex-valued MR images. Working in the complex domain instead of the amplitude one allows us to consider the data bivariate Gaussian distributed, and thus to implement a simple Least Square (LS) estimator on the available complex data. The proposed estimator results to be simple, accurate and unbiased.

## 1. Introduction

Magnetic Resonance Imaging (MRI) is a technique used in medical environment to produce high quality images of tissues inside human body. MRI is based on the principles of nuclear magnetic resonance (NMR), a spectroscopic technique used to obtain microscopic chemical and physical information about molecules. In MRI, a radiofrequency signal stimulates the hydrogen atoms of an object posed in a uniform magnetic field to emit complex-valued signals. These signals are coherently collected by MR coil, converted from analog to digital and recorded in the so called *k-space*, providing MR raw data. Commonly known MR images are defined in the spatial domain, obtained by Fourier transforming the raw data [1,2].

MR images are characterized by the combination of three intrinsic parameters: the spin density of hydrogen atoms *ρ*, the spin-lattice relaxation time T_1_ and the spin-spin relaxation time T_2_ [2]. In particular, relaxation times can give useful information about local environment interaction and provide a major source of contrast in MR images, while, a quantitative analysis of spin-spin relaxation time T_2_ can give useful information for cancer discrimination [3].

Conventional relaxation parameter estimation techniques work on the amplitude of MR images. A commonly used approach consists in using Least Square (LS) estimator [4]. This approach assumes additive Gaussian distributed noise on amplitude MR images. However, the noise probability density function (pdf) follows the Rice distribution, as amplitude MR images are computed from two independent Gauss distributed random processes (the real and imaginary parts) [5–7]. As far as we know, many papers present in literature consider the Rice distribution for the amplitude MR images [8–12], but only two papers, [13] and [14], have considered it for the T_2_ estimation problem. In the first one [13], although assuming Rice distribution for the model, the authors estimate spin-spin relaxation parameter still using LS. They justify the use of LS by stating that at high SNRs Rice distribution approaches a Gaussian pdf. This assumption, however, introduces a bias in the estimation, especially for low SNRs. In [14], the authors propose a Maximum Likelihood Estimator (MLE) for retrieving relaxation parameters. Since the correct noise model is assumed, the proposed estimator is able to avoid the bias even at low SNRs in case of large data sets.

In this paper we propose an approach for spin-spin relaxation time estimation that works directly on the complex-valued MR images instead of amplitude. Working in complex domain allows us to implement LS estimation since the noise is Gaussian both on real and imaginary parts. Differently from [13], we do not expect the estimator to be biased since we use the correct model. Compared to [14], the proposed approach has two main advantages: first of all we exploit twice the available data, as the estimation is performed using the real and the imaginary parts. This allows us to use more information to reduce noise obtaining a more accurate estimation. Secondly, LS estimation ensures lower computational cost compared to the Rice based MLE proposed in [14]. Note that the likelihood function in Rice case contains the Bessel function, which can be harder to be managed than an exponential function.

In Section 2 the statistical model is briefly addressed. In Section 3 the accuracy of the proposed model is evaluated exploiting Cramer Rao Lower Bounds (CRLB) and a comparison with other models present in literature is discussed. The performances of the proposed estimator are shown in Section 4. In Section 5, a fast version of the Non Linear LS estimator is proposed. Finally, we draw some conclusions about the presented technique.

## 2. Statistical Description of MR Images

In MRI the data are recorded in the *k-space*, where they are corrupted by additive, zero mean and uncorrelated Gaussian noise samples [15]. In order to obtain MR images in spatial domain, an inverse Fourier transform is required. Thanks to the linearity and orthogonality of this operation, complex-valued MR images are still corrupted by additive, zero mean and uncorrelated Gaussian noise in both real and imaginary parts. After the inverse Fourier transform, although the signal intensity and the noise variance could be modified, the ratio between the signal power and the noise power does not change.

Let us consider a pixel of a complex-valued MR image; in a noise free case, the signal expression is given by:

(1) $$y_{0}=me^{i\phi }$$

where *m* represents the amplitude of the signal and *ϕ* the phase. The amplitude *m* depends on the used imaging sequence and on the unknown parameters **θ** (the spin density of hydrogen atoms *ρ*, the spin-lattice relaxation time T_1_ and the spin-spin relaxation time T_2_) *via*:

(2) $$m=f(\theta )=f\left(\rho ,T_{1},T_{2}\right)$$

while the phase *ϕ* depends on the local intensity of the magnetic field (field map) [16][17].

Equation (1) can be alternatively written in terms of real and imaginary parts:

(3) $$y_{0}=y_{0R}+i\cdot y_{0I}=m\text{cos}(\phi )+i\cdot m\text{sin}(\phi )=f(\theta )\text{cos}(\phi )+i\cdot f(\theta )\text{sin}(\phi )$$

Let us now focus on the noisy case. As stated before, real (*y_R_*) and imaginary (*y_I_*) parts are corrupted by additive, zero mean and uncorrelated Gaussian noise *w_R_* and *w_I_*:

(4) $$\begin{array}{l} y_{R}=f(\theta )\text{cos}(\phi )+w_{R}\\ y_{I}=f(\theta )\text{sin}(\phi )+w_{I} \end{array}$$

providing the following distributions:

(5) $$\begin{array}{l} P_{Y_{R}}\left(y_{R}\right)=\frac{1}{\sqrt{2\pi \sigma^{2}}}e^{-\frac{{\left(y_{R}-f(\theta )\text{cos}(\phi )\right)}^{2}}{2\sigma^{2}}}\\ P_{Y_{I}}\left(y_{I}\right)=\frac{1}{\sqrt{2\pi \sigma^{2}}}e^{-\frac{{\left(y_{I}-f(\theta )\text{sin}(\phi )\right)}^{2}}{2\sigma^{2}}} \end{array}$$

where *σ*^2^ represents noise variance. By multiplying the two equations (5), we obtain the joint statistical distribution The proposed method is based on this statistical distribution of the measured data, called Gaussian Complex Model.

Other techniques work on the amplitude instead of the complex domain, assuming different distribution for the data [14]. In particular, starting from Equation (5), it is easy to show that the amplitude is corrupted by Rice distributed noise [5–7], leading to the following distribution:

(6) $$P_{M}(\hat{m})=\frac{\hat{m}}{\sigma^{2}}e^{\left(-\frac{{\hat{m}}^{2}+f^{2}(\theta )}{2\sigma^{2}}\right)}I_{0}\left(\frac{f(\theta )\hat{m}}{\sigma^{2}}\right)$$

where *I*_0_(·) is the modified Bessel function of the first kind with order zero and *m̂* represents the measured noisy amplitude.

The term *f*(**θ**) is determined by the imaging sequence adopted during the MR scan. In this paper we consider a conventional spin echo imaging sequence which leads to [2]:

(7) $$f(\theta )=\rho \cdot e^{-\frac{T_{E}}{T_{2}}}\cdot \left(1-e^{-\frac{T_{R}}{T_{1}}}\right)$$

where the instrumental variables T*_E_* and T*_R_* are the Echo Time and the Repetition Time of the sequence, respectively.

Since we are interested in T_2_ estimation, we enclose T_1_ and *ρ* in a new parameter *k*, called pseudodensity, as in [13]. So *f*(**θ**) becomes:

(8) $$f(\theta )=k\cdot e^{-\frac{T_{E}}{T_{2}}}$$

which is commonly referred as monoexponential transversal magnetization decay model and is widely adopted [13,14]. In the next Section the achievable accuracy for T_2_ estimation using the proposed model is addressed exploiting CRLB.

## 3. Cramer Rao Lower Bounds Evaluation

The Cramer Rao Lower Bounds provide the minimum variance for a given acquisition model that any unbiased (non polarized) estimator can reach [18]. They provide a benchmark against which we can compare the performances of any unbiased estimator. Moreover, they alert us of the physical impossibility of finding an unbiased estimator with a variance smaller than the bounds. CRLB for amplitude estimation in MR Imaging have been presented in different papers [8,9]. In this section, a study on the CRLB for the specific T_2_ estimation problem using the Gaussian Complex Model is conducted. To assess the performances of the proposed model, the CRLB are numerically evaluated using Monte Carlo method.

In the following simulation, a pixel with *k* = 80 and T_2_ = 100 msec is considered, corresponding to white matter tissue at 1.5 Tesla. The number of Monte Carlo iterations is fixed to 10^5^.

**Figure 1.**
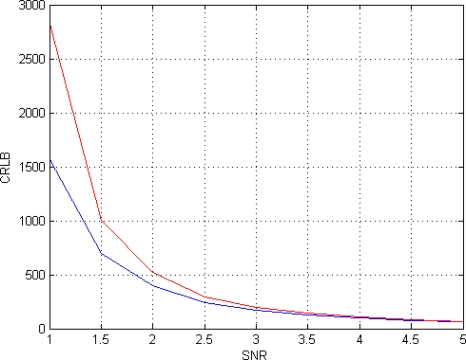
CRLB behavior in case of different SNRs for the proposed Gaussian Complex Model (blue) and for the Rice Amplitude Model (red).

First of all the dependency of CRLB on SNR is analyzed. SNR is defined as the ratio between the mean value of *f*(**θ**) and noise standard deviation *σ*. A set of eight T*_E_* equispaced in the interval [50 350] msec and a phase *ϕ* = 60° are considered. The CRLB for this configuration are presented in the second column of Table 1; for comparison, in the third column the CRLB for the same configuration but using the amplitude model (Equation (6)) are reported. The CRLB behaviors for both acquisition models are also presented in Figure 1.

Investigating the behavior of CLRB in Table 1 and in Figure 1, it can be noted that the Gaussian complex model (5) is more accurate than the Rice amplitude (6) one, in particular for low SNRs. As a matter of fact, for the considered SNR values range (SNR = [1–5]), the Minimum Variance Unbiased (MVU) estimator for the Rician model cannot reach the accuracy of the MVU estimator for the Gaussian case. For larger SNRs, the two models tend asymptotically to ensure the same accuracy.

**Figure 2.**
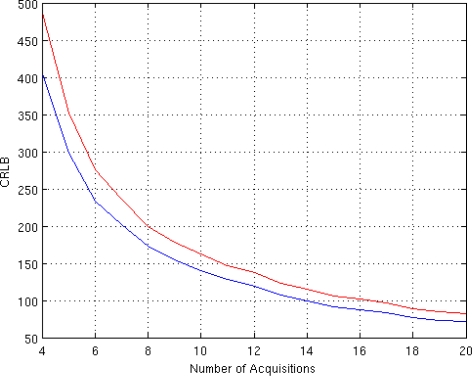
CRLB behavior in case of different number of acquisitions for the proposed Gaussian Complex Model (blue) and for the Rice Amplitude Model (red).

The dependency of CRLB on the number of available acquisition (*i.e.*, the number of T*_E_*) is then analyzed. The T*_E_* values are equispaced in the interval [50 350] msec. The SNR is set to 3 and the phase *ϕ* equal to 60°. The CRLB behaviors for both acquisition models are presented in Figure 2.

Investigating the behavior of CLRB in Figure 2, it can be noted that the number of acquisition has the same impact on both models. The behavior of the CRLB for the two considered models is the same, except for an offset. This offset is representative of the better accuracy achievable by the Gaussian model.

The CRLB evaluation can be useful not only to compare the achievable accuracy of different models, but also to investigate the best parameter configuration for a considered model.

**Figure 3.**
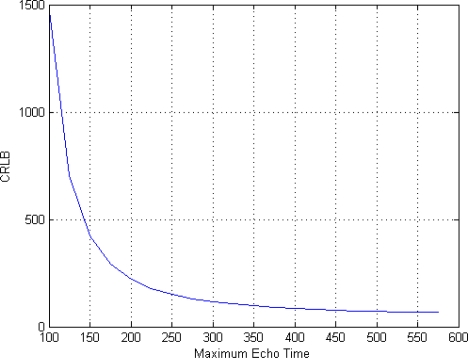
Impact of different spacing between TE values on the CRLB.

In order to evaluate the impact of T*_E_* values on the CRLB we perform two more analysis, using SNR = 4, *ϕ* = 60° and eight acquisitions (*i.e.*, eight T*_E_* values). The first one is related to the role of different spacing between T*_E_* values. Such values are chosen in the interval [50 T*_E__max*] msec, varying T*_E__max*. The results of this simulation are shown in Figure 3. As expected, increasing T*_E__max* (*i.e.*, increasing the spacing between T*_E_* values) has a positive effect on the achievable accuracy. Anyway, saturation appears when T*_E__max* assumes high values.

**Figure 4.**
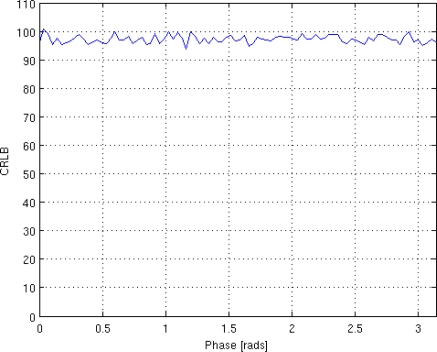
Impact of *ϕ* on the CRLB.

Finally, the influence of phase *ϕ* on the CRLB is investigated. The simulation has been conducted considering eight T*_E_* values equispaced in the interval [50 350] msec, SNR = 4. The results, shown in Figure 4, indicate that *ϕ* does not substantially affect the behaviour of CRLB.

## 4. Non Linear Least Square Estimator: Results and Discussion

In this section, we present the estimator for the proposed model and we evaluate its performances in terms of mean and variance, compared to the CRLB, for different case studies.

Since we are working in the complex domain, as previously stated, signal statistical distributions for both real and imaginary parts are Gaussian. This assumption allows us to use a simple Least Square estimator. In particular we implement a Non Linear LS (NLLS) as the signal is characterized by an exponential decay Equation (8). The NLLS is obtained by minimizing the square difference between the observed data and equation (8) which contains the unknown parameter.

The presented results are obtained applying the method to realistically simulated data sets, using the same parameters of the previous section.

**Figure 5.**
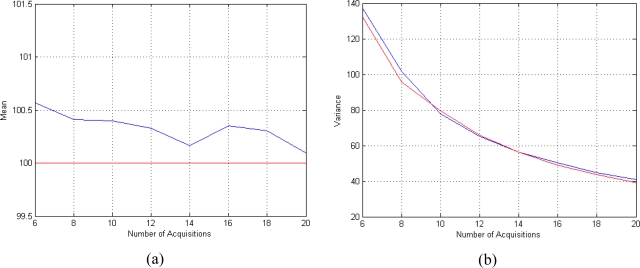
Performances of the T_2_ estimator for different number of available complex-valued data. (a) Mean behavior (the mean is plotted in blue and the true value is plotted in red) and (b) variance behavior (the variance is plotted in blue and the CRLB are plotted in red). The results are obtained using SNR = 4, *ϕ* = 60° and *N* T*_E_* values equispaced in the interval [50 350] msec.

In the first case study the performances of the proposed estimator for different number of available complex-valued data sets *N* are evaluated. Figure 5(a) shows the trend of the mean value of the T_2_ estimator for different number of T*_E_*. As we can see the estimator results non-polarized since the bias is not present already with few data. Figure 5(b) shows the behaviour of the variance of the T_2_ estimator, compared to the CRLB. It can be noted that the estimator rapidly tends to be efficient, since its variance approaches the CRLB as the number of data grows.

**Figure 6.**
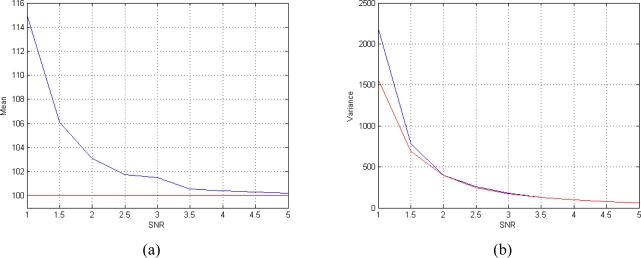
Performances of the T2 estimator for different SNRs. (a) Mean behavior (the mean is plotted in blue and the true value is plotted in red) and (b) variance behavior (the variance is plotted in blue and the CRLB are plotted in red). The results are obtained using *ϕ* = 60° and N = 8 TE values equispaced in the interval [50 350] msec.

For the second case study, we consider the performances of the T_2_ estimator for different values of SNR. We fix the number of acquisitions to 8 (*N* = 8). The performances of the estimator are satisfactory even at low SNRs. As it is shown in Figures 6(a) and (b) the mean of the estimator tends to the true value and its variance approaches the CRLB already at low SNRs.

Finally the proposed algorithm has been tested on a 256 × 256 realistically simulated slice of the human head. The spin density and the relaxation times of the simulated tissues are reported in Table 2, according to the Shepp-Logan phantom proposed in [19]. The pseudodensity values *k* have been obtained considering T*_R_* = 5,000 msec.

**Figure 7.**
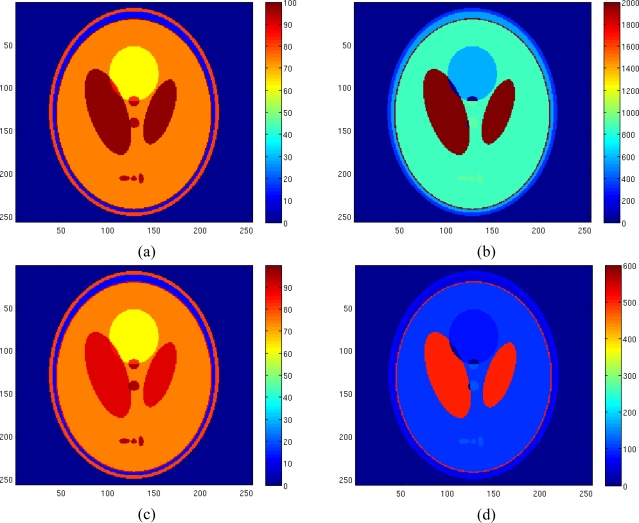
Reference spin density map (a), reference T_1_ map (b), reference pseudodensity map (c), reference T_2_ map (d).

Figure 7 shows the true spin density, the pseudodensity and the relaxation times maps of the considered tissues. Starting from these maps and fixing 8 T*_E_* values equispaced in the interval [50 350] msec, 8 complex-valued images have been generated. Phase value has been set to 60° and noise has been added. The noise variance has been set equal to 3.5.

**Figure 8.**
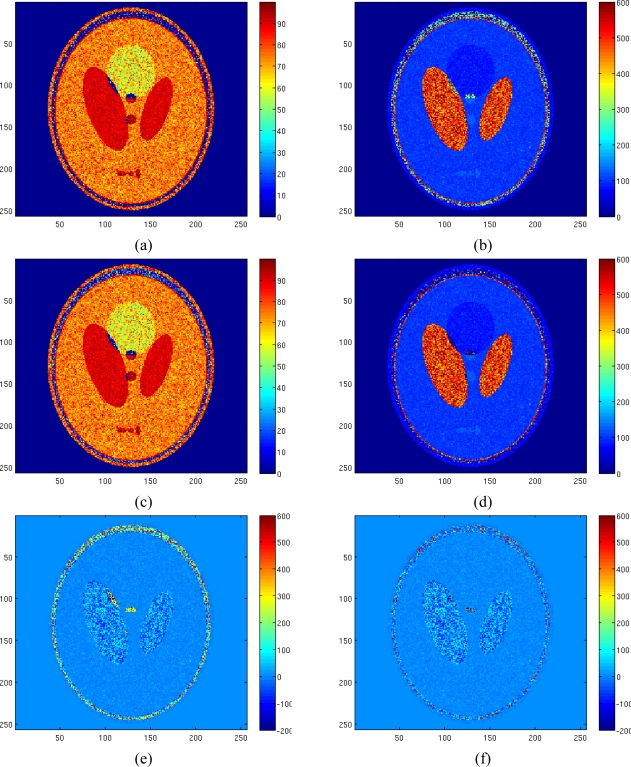
Estimated pseudodensity map using ML estimator with Rician amplitude model (a), estimated T_2_ map using ML estimator with Rician amplitude model (b), estimated pseudodensity map using LS estimator with Gaussian complex model (c), estimated T_2_ map using LS estimator with Gaussian complex model (d), T_2_ reconstruction error map using ML estimator with Rician amplitude model (Normalized mean square error equal to 0.1546) (e), T_2_ reconstruction error map using LS estimator with Gaussian complex model (Normalized mean square error equal to 0.1029) (f).

The results of the ML estimator applied to the Rician amplitude model and of the NLLS estimator with Gaussian complex model are reported in Figures 8(a) and (b) and Figures 8(c) and (d), respectively. In order to better appreciate the obtained results the reconstruction error map using both estimators have been reported in Figure 8(e) and (f). All no signal areas have been masked after estimation. The simulation demonstrates the effectiveness of the proposed method and its better accuracy compared to the other considered method.

Concerning the computational cost of the algorithm, we have to underline that an optimization algorithm needs to iteratively compute a function to find the solution. Clearly, Gaussian Complex Model (Equation (5)) shows a lower computational complexity compared to a Rician one (Equation (6)), where the Bessel function has to be evaluated. This leads to a higher computational time for optimizing Rician distribution, which in some applications could be a problem. For the considered simulation, computational times were 660 sec for the LS estimator applied to the Gaussian model and 1,900 sec for the ML estimator applied to the Rician model using a SUN Ultra 40 workstation.

**Figure 9.**
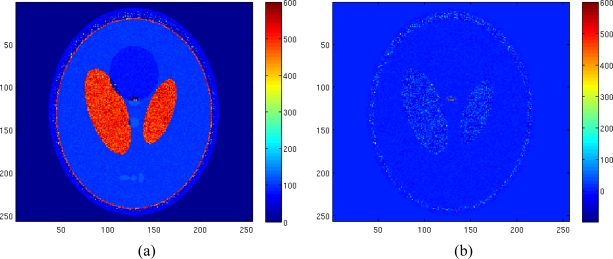
Estimated T_2_ map using LS estimator with Gaussian complex model in case of *σ* = 1.5 (a), T_2_ reconstruction error map (Normalized mean square error equal to 0.0210) (b).

Figure 9 shows the reconstruction of the T2 map and the reconstruction error map for the same data set of Figure 7, but with a noise variance of 1.5, showing the accuracy of the proposed method also with less noisy data; with this noise variance, the computation of the Bessel function in the Rician pdf becomes a not trivial task.

The better accuracy of the Gaussian Complex Model respect to the Rician one can be explained in terms of functions to be optimized. In the first case, the square error function between the observed data and (7) (Least Square) has to be minimized. In the second one, the likelihood function, obtained by pdf (6) after having observed the data, has to be maximized.

**Figure 10.**
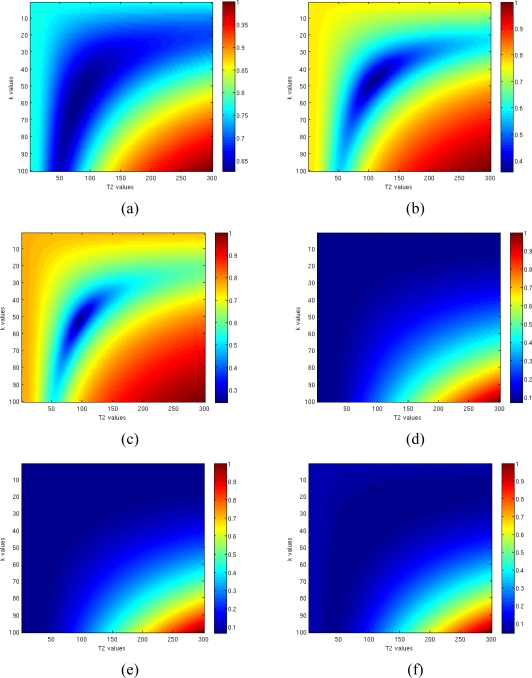
Behaviors of normalized functions to be minimized in case of Gaussian Complex Model (a–c) and Rician model (e–f) for SNR = 1 (a,d), SNR = 3 (b,e) and SNR = 5 (c,f).

Figure 10 shows the normalized functions to be minimized in the Gaussian Complex Model square error function–Figure 10(a), 10(b) and 10(c)] and in the Rician model [opposite likelihood function—Figure 10(d)–(f)] simulating a pixel with k = 50 and T2 = 100, and considering eight TE values. As expected, changing the SNR from 1 [Figure 10(a) and 10(d)] to 3 [Figure 10(b) and 10(e)] and to 5 [Figure 10(c) and 10(f)] has a positive effect on global minimum, allowing an easier minimization. Anyway in all cases the minimum related to the Rician model can be found with a lower accuracy respect to the Gaussian model case. This is due to the behavior of likelihood function (6) which has a flat surface in the proximity of the minimum: even increasing the SNR from 1 to 5 the absolute minimum still remains located in a large and flat area.

## 5. Fast Non Linear Least Square Estimator

In Section 3 we presented the comparison between the Amplitude/Rice and Complex/Gaussian models, showing the best performances of the second one in both estimation accuracy and computational time. In this section we introduce a method to improve the speed of the second approach, making it quasi real time. This approach is based on Non Linear LS and is defined in [18]:

(9) $${\hat{T}}_{2}=\text{arg}\;\text{min}\;y^{T}H\left(T_{2}\right){\left(H^{T}\left(T_{2}\right)H\left(T_{2}\right)\right)}^{-1}H^{T}\left(T_{2}\right)y$$

where **y** = [*y*_1_*_R_*, *y*_1_*_I_*, *y*_2_*_R_*, *y*_2_*_I_*,…*y_NR,_ y_NI_*]^T^ is the collection of the real and imaginary parts of measured data for the *N* T*_E_* values [T*_E_*_1_, T*_E_*_2_*, …, T_EN_*] ^T^, and:

(10) $$H\left(T_{2}\right)={\left[\begin{array}{lllllll} e^{-\frac{T_{E1}}{T_{2}}} & e^{-\frac{T_{E1}}{T_{2}}} & e^{-\frac{T_{E2}}{T_{2}}} & e^{-\frac{T_{E2}}{T_{2}}} & \cdots & e^{-\frac{T_{\mathrm{EN}}}{T_{2}}} & e^{-\frac{T_{\mathrm{EN}}}{T_{2}}} \end{array}\right]}^{T}$$

where the components of **H**(T_2_) are reported twice in order to take into account both real and imaginary parts of **y**.

**Figure 11.**
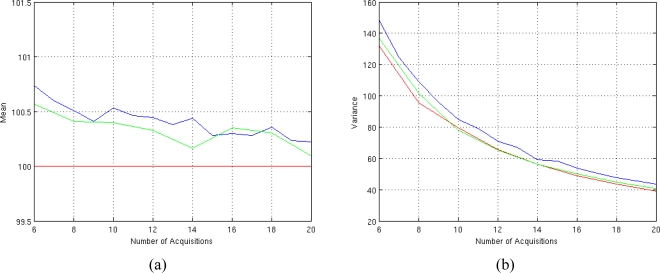
Performances of the Fast T_2_ estimator for different number of available complex-valued data. (a) Mean behavior (the mean of Fast Non Linear LS estimator is plotted in blue, the mean of the classic Non Linear LS estimator is plotted in green and the true value is plotted in red) and (b) variance behavior (the variance of Fast Non Linear LS estimator is plotted in blue, the variance of the classic Non Linear LS estimator is plotted in green and the CRLB value is plotted in red). The results are obtained using SNR = 4, *ϕ* = 60° and *N* T*_E_* values equispaced in the interval [50–350] msec.

The estimator of Equation (9) allows us to perform a monodimensional minimization instead of a bidimensional one, greatly reducing the computational time. For example, spin-spin relaxation time estimation of 64 × 64 pixel image using eight acquisitions is performed in 71 seconds for classic Non Linear LS and in 10 seconds for Fast Non Linear LS estimator Equation (9) with our workstation. Figures 11(a) and (b) show the performances of this estimator varying the number of T*_E_* values.

As we can see, in terms of performances, the fast estimator can be considered unbiased as the classic one while the accuracy is slightly worst compared to the classic one. This is due to the non linear relation between the observation matrix **H** and T_2_ in equation (10). Anyway this loss of accuracy is balanced by the 7× speed improvements in computational time which can be very useful for quasi real time applications.

A final note about the possibility of estimating T_1_ has to be marked. In the proposed approach we are interested in the estimation of T_2_. Thus all our simulations and analysis are conducted considering only the spin-spin relaxation parameter. Anyway, it is easy to show that the proposed approach can be extended also for the estimation of T_1_, expliciting Equation (7) and (8) in terms of spin-lattice relaxation parameter instead of spin-spin relaxation parameter.

## 6. Conclusions

In this paper a new statistical technique for the estimation of relaxation time parameters in Magnetic Resonance Imaging is presented. Differently from other models present in literature, we propose to work directly with real and imaginary parts of complex-valued Magnetic Resonance images. This implies to consider the acquired data corrupted by additive, zero mean, uncorrelated complex-valued Gaussian noise samples, allowing us to use a simple Non Linear LS estimator to retrieve the unknown parameter T_2_ without introducing any bias in the estimation. The evaluation of CRLB let us state that the proposed model is able to reach a better accuracy compared to other models. Moreover, the conducted simulations show the good performances of the proposed Non Linear LS estimator: the estimator is unbiased and rapidly tends to be efficient. Finally a fast Non Linear LS estimator has been proposed, providing an algorithm for quasi real time applications. The next step of this work will be the application of the approach to real data sets.

## Figures and Tables

**Table 1.** CRLB values in case of different SNRs for the proposed Gaussian Complex Model and for the Rice Amplitude Model.

SNR	Gaussian Complex Model	Rice Amplitude Model	Relative Difference
1	1570.5	2836.1	+80.6%
2	399.3456	520.6266	+30.3%
3	172.1815	199.2489	+15.7%
4	97.0954	107.6980	+10.9%
5	63.0583	67.1536	+6.5%

**Table 2.** Spin density and relaxation times for the Shepp-Logan phantom. The *k* values have been obtained using T*_R_* = 5,000 msec.

Tissue	*ρ*(%)	T_1_ (msec)	*k*	T_2_ (msec)
Scalp	80	324	80	70
Bone	12	533	11.99	50
CSF	98	2,000	89.95	500
Gray Matter	74.5	857	74.28	100
White Matter	61.7	583	61.68	80
Tumor	95	926	94.57	120

## References

[1] Slichter P. (1996). Principles of Magnetic Resonance.

[2] Cho Z.-H., Jones J.P., Singh M. (1993). Foundations of Medical Imaging.

[3] Roebuck J.R., Haker S.J., Mitsouras D., Rybicki F.J., Tempany C.M., Mulkern R.V. (2009). Carr-Purcell-Meiboom-Gill imaging of prostate cancer: quantitative T2 values for cancer discrimination. Magn. Reson. Imaging.

[4] Liu J, Nieminen A., Koenig J.L. (1989). Calculation of T1, T2 and proton spin density in nuclear magnetic resonance imaging. J. Magn. Resonance.

[5] Leon-Garcia A. (1994). Probability and Random Processes for Electrical Engineering.

[6] Rice S.O. (1994). Mathematical analysis of random noise. Bell Syst. Technol. J.

[7] Gudbjartsson H., Patz S. (1995). The Rician distribution of noisy MRI data. Magn. Reson. Medicine.

[8] Sijbers J., den Dekker A.J. (2004). Maximum Likelihood estimation of signal amplitude and noise variance from MR data. Magn. Reson. Medicine.

[9] Rowe D.B. (2005). Parameter estimation in the magnitude-only and complex-valued fMRI data models. Neuroimage.

[10] Rowe D.B., Logan B.R. (2005). Complex fMRI analysis with unrestricted phase is equivalent to a magnitude-only model. Neuroimage.

[11] Zhu H., Li Y., Ibrahim J.G., Shi X., An H., Chen Y., Lin W., Rowe D.B., Peterson B.S. (2009). Rician regression models for magnetic resonance images. J. Am. Stat. Assoc.

[12] Koay C.G., Basser P.J. (2006). Analytically exact correction scheme for signal extraction from noisy magnitude MR signals. J. Magn. Resonance.

[13] Bonny J.M., Zanca M, Boire J.Y., Veyre A. (1996). T2 maximum likelihood estimation from multiple spin-echo amplitude images. Magn. Reson. Med.

[14] Sijbers J., den Dekker A.J., Raman E, Van Dyck D. (1999). Parameter estimation from amplitude MR images. Int. J. Imag. Syst. Technol.

[15] Wang Y., Lei T., Sewchand W., Mun S.K. (1996). MR imaging statistics and its application in image modelling. Proc SPIE Conf. Med. Imaging.

[16] Jezzard P., Balaban R.S. (1995). Correction for geometric distortion in echo planar images from B_0_ field variations. Magn Reson Med.

[17] Baselice F., Ferraioli G., Shabou A. (2010). Field Map Reconstruction in Magnetic Resonance Imaging Using Bayesian Estimation. Sensors.

[18] Kay S.M. (1993). Fundamentals of Statistical Signal Processing, Estimation Theory.

[19] Gach M.H., Tanase C., Boada F. 2D & 3D Shepp-Logan Phantom Standards for MRI.

